# Mechanisms behind gender transformative approaches targeting adolescent pregnancy in low- and middle-income countries: a realist synthesis protocol

**DOI:** 10.1186/s13643-024-02513-4

**Published:** 2024-03-23

**Authors:** Shruti Shukla, Ibukun-Oluwa Omolade Abejirinde, Sarah R. Meyer, Yulia Shenderovich, Janina Isabel Steinert

**Affiliations:** 1 TUM School of Social Sciences and Technology, Technical University of Munich, München, 80333 Germany; 2 Division of Social and Behavioural Health Sciences, University of Toronto Dalla Lana School of Public Health and Women’s College Hospital Research Institute, Toronto, Ontario Canada; 3 Institute for Medical Information Processing, Public Health and Health Services Research, Ludwig-Maximilians-Universität München, Biometry, and Epidemiology, Munich, Germany; 4 Centre for Development, Evaluation, Complexity and Implementation in Public Health Improvement (DECIPHer), School of Social Sciences, Wolfson Centre for Young People’s Mental Health, Cardiff University, Cardiff, UK; 5 Department of Social Policy and Intervention, University of Oxford, Oxford, UK

**Keywords:** Adolescent girls, Gender norms, Adolescent pregnancy, Realist methodology, Gender transformative approaches

## Abstract

**Introduction:**

Adolescent pregnancy is defined as pregnancy at the age of 19 or below. Pregnancy and childbirth complications are the most significant cause of death among 15–19-year-old girls. Several studies have indicated that inequitable gender norms can increase the vulnerability of adolescent girls, including violence exposure, early marriage, and adolescent pregnancy. To address these disparities, gender transformative approaches aim to challenge and transform restrictive gender norms, roles, and relations through targeted interventions, promoting progressive changes. This realist review aims to synthesise existing evidence from a broad range of data sources to understand how, why, for whom, and in what contexts gender transformative approaches succeed in reducing adolescent pregnancy in low- and middle-income countries.

**Method and analysis:**

We employ a five-step realist synthesis approach: (1) clarify the scope of review and assessment of published literature, (2) development of initial programme theories, (3) systematic search for evidence, (4) development of refined programme theories, and (5) expert feedback and dissemination of results. This protocol presents the results of the first three steps and provides details of the next steps.

We extracted data from 18 studies and outlined eight initial programme theories on how gender transformative approaches targeting adolescent pregnancy work in the first three steps. These steps were guided by experts in the field of sexual and reproductive health, implementation science, and realist methodology. As a next step, we will systematically search evidence from electronic databases and grey literature to identify additional studies eligible to refine the initial programme theories. Finally, we will propose refined programme theories that explain how gender transformative approaches work, why, for whom, and under which circumstances.

**Ethics and dissemination:**

Ethics approval is not required because the included studies are published articles and other policy and intervention reports. Key results will be shared with the broader audience via academic papers in open-access journals, conferences, and policy recommendations. The protocol for this realist review is registered in PROSPERO (CRD42023398293).

**Supplementary Information:**

The online version contains supplementary material available at 10.1186/s13643-024-02513-4.

## Background

Adolescent health has become a priority on the health policy agenda in recent years. Guidelines ranging from the United Nations high-level meeting on youth in 2010 to the Global Strategy for Women’s, Children’s, and Adolescents’ Health 2016–2030 to the most recent 1.8 Billion Young People for Change Campaign in 2023 have highlighted the growing attention to adolescents [1, 2]. Adolescence is a critical period of transition from childhood to adulthood characterised by changes in cognitive, physical, social, and sexual capabilities [3]. These changes are often accompanied by heightened gender inequality and restrictive social norms, which primarily impact adolescent girls and are associated with heightened risk for violence exposure, sexually transmitted infections, early marriage, and adolescent pregnancy [1, 4].

Adolescent pregnancy is defined as pregnancy at the age of 19 or below [5]. While the global levels of adolescent birth rate have declined from 64.5 births per 1000 women in 2000 to 42.5 births per 1000 women in 2021, it is still an important health indicator of the Sustainable Development Goals (SDGs) [6]. As of 2019, about 21 million adolescent girls aged 15–19 became pregnant in low- and middle-income countries (LMICs), 12 million of whom gave birth [7]. A recent study found that sub-Saharan Africa, followed by Latin America and the Caribbean, and South and Southeast Asia had the highest prevalence of adolescent pregnancies [8]. A significant consequence of adolescent pregnancy is the high risk of adverse health outcomes for both adolescent mothers and their children, mainly because of complications during pregnancy and childbirth, limited access to prenatal care, and a higher likelihood of living in poverty for adolescent mothers [9, 10]. Adolescent mothers may suffer from eclampsia, puerperal endometritis, and systemic infections, and their children might be at risk of low birthweight, preterm birth, severe neonatal conditions, and newborn mortality [5, 11, 12]. Early marriage, lack of sex education and health services, poor socioeconomic background, and sexual risk behaviours are vital predictors of the high prevalence of adolescent pregnancies in LMICs [13]. In recent years, the COVID-19 pandemic has caused additional disruption to access to family planning services and education and also led to a surge in gender-based violence [14–18], which are known correlates for adolescent pregnancy. For instance, World Vision estimated that school closures alone could lead to a 65% increase in adolescent pregnancies and may block one million girls in sub-Saharan Africa from returning to school [14]. Another study from this region highlights that inequitable gender norms are associated with adolescent pregnancy [19]. The lower value of education and prioritisation of household chores for girls, men’s control over contraceptive use, lower social status, and lack of decision-making power in the daily lives of adolescent girls are some of the vital gender norms surrounding this association [19, 20]. Therefore, it is essential to address these restrictive norms to ensure better health outcomes for adolescents throughout their life course.

One way to address restrictive gender norms is the application of *gender transformative approaches* (GTAs) to programme design to explicitly examine and address power relations associated with men and women and boys and girls in programmes and interventions [21]. This approach differs from the gender blind (ignores the power dynamics) and gender accommodating (acknowledges differences without addressing root causes) approaches by challenging the root causes of gender inequality and reshaping unequal power relations [22]. Levy et al. (2020) systematically reviewed the characteristics of successful programmes targeting gender inequality and restrictive gender norms. The review found that these programmes can improve knowledge, attitudes, and behaviours around health among children, adolescents, and young adults. While these are essential impacts, there are significant gaps in evidence on outcomes beyond knowledge and attitudes. For instance, only 5 out of 61 evaluation studies included in the review measured changes in the incidence of unwanted or unintended pregnancies [20]. Furthermore, most of the included studies were quantitative and often implemented interventions only with girls, thus limiting our understanding of the specific mechanisms of change and how they may differ for girls and boys. Specific to adolescent pregnancy, a few existing systematic reviews suggest that knowledge-based or skill-based interventions, contraceptive-promoting interventions, conditional cash transfers, and programmes lowering barriers to education could potentially reduce adolescent pregnancies [23–25]. However, evidence on these programmes is outdated, given the rise of adolescent pregnancy amidst the COVID-19 pandemic. Furthermore, authors often did not discuss potential mechanisms of change leading to a reduction in adolescent pregnancy. Lastly, these reviews and included studies do not measure gender or social norms change or shifts in norms and how that impacts adolescent pregnancy. These gaps in research point to the importance of exploring the underlying contexts and mechanisms that contribute to the potential success of GTAs targeting adolescent pregnancy.

This review employs a realist approach to fill the above-mentioned research gaps. It will investigate how, why, for whom, and in what contexts gender transformative approaches succeed in reducing adolescent pregnancy. We aim to synthesise existing evidence from a broad range of data sources to develop programme theories to explain how contextual factors, intervention strategies, and programme mechanisms of GTAs may influence adolescent pregnancy.

## Methodology

Realist synthesis is a theory-driven approach to evidence synthesis based on the philosophical principles of realism. Realism is defined as a broad logic of inquiry that sits between positivism and constructivism and agrees that social reality cannot be measured directly [26]. However, it can be understood by examining the relationship between context and outcome, underlined by causal forces (mechanisms) in which events occur and the outcomes produced [26]. A realist review is concerned with answering how an intervention works, whom it works for, and in what circumstances it works [27]. It differs from the traditional systematic review in that it emphasises the importance of contextual factors in shaping the effectiveness of interventions and their associated mechanisms. A realist review involves an iterative process of theory building and testing — which includes building an initial programme theory based on preliminary data and then refining this theory based on additional qualitative and quantitative data.

Gender transformative approaches are both ontologically and epistemologically complex [28]. GTAs work by changing participants’ decision-making process and altering the resources and opportunities available to them within a specific context by eliciting certain mechanism(s). Therefore, it is essential to unpack ‘what works’ to explain these interactions. This review aims to achieve this by actively seeking out the contextual (C) factors that are hypothesised to have triggered the relevant mechanism (M) to generate the outcome (O) of interest [26]. In a realist review, secondary data is used to develop these CMO configurations (CMOCs), categorise them into theory-driven initial programme theories (IPTs), and then test (confirm, refute, or refine) them with additional data to produce the refined programme theories [29].

**Fig. 1:**
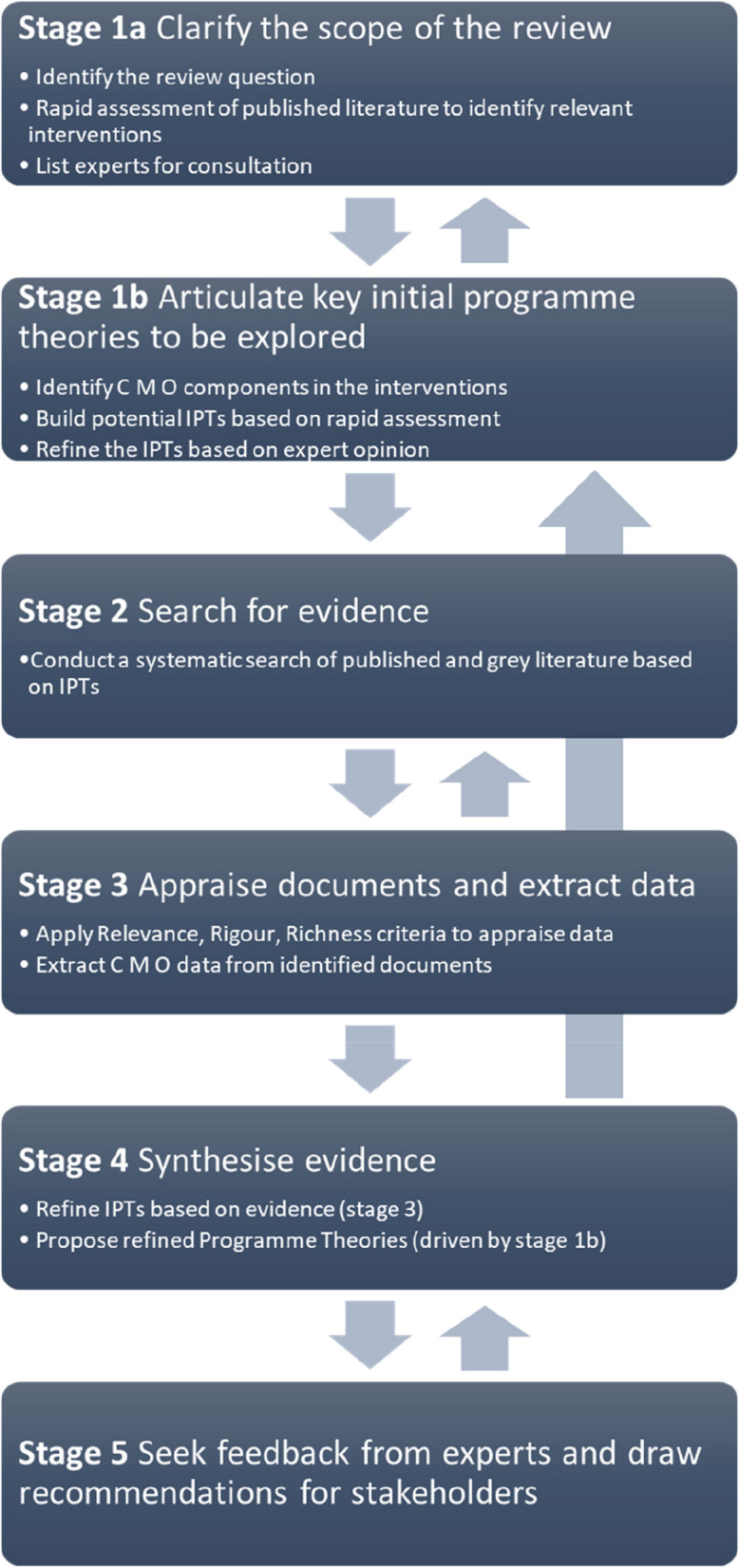
Steps of realist review

A programme theory is an idea about how the programme works, i.e. if we do X, then Y will happen because of Z [27]. Through this ideation process, we observe patterns in published literature and develop plausible theories that inform the programme design and implementation in different settings [30]. In this realist review, we operationalise mechanisms as resources and reasoning. Resources refer to strategies or components introduced by the interventions in a specific context to elicit change. Reasoning refers to the behavioural response of participants triggered by these resources [31]. Such an operationalisation helps differentiate if data contributes contextually or mechanistically. We will utilise CMO configurations organised as *if…then…because* statements to answer the following research question: *What are the underlying contexts and mechanisms that lead to the success of gender transformative approaches in reducing adolescent pregnancy in low- and middle-income countries?* We will follow the iterative process described by Pawson et al. (2005) to conduct our realist synthesis review [28]. While we follow the five key steps outlined in the article, we also adapt the sub-steps to fit our purpose (Fig. 1). The protocol for this realist review is registered in PROSPERO (CRD42023398293), and we will follow the RAMESES publication standards for this realist synthesis [32].

### Stages 1a and 1b — Clarify the scope of the review and development of initial programme theories

The research question for this review was developed as a part of the first author’s (S. S.) doctoral study proposal. As a next step, to refine the purpose of the review, we conducted a rapid assessment of seven systematic reviews on gender transformative approaches targeting adolescent health [20, 22, 24, 33–36]. This search helped build the first list of interventions for developing the IPTs. We identified 18 studies from the seven systematic reviews and used them for IPTs formation. A codebook based on the CMO components was developed to guide data extraction to identify relevant factors contributing to the IPTs [37]. One author (S. S.) designed the codebook, and two other authors revised it in an online discussion (S. M., I. O. A.). The codebook is provided in Additional Table 1. A list of experts in the field of sexual and reproductive health (SRH), programme design, realist evaluation, and adolescent health was prepared based on identified literature. We approached 12 experts via mail for their involvement in the feedback round, 7 of whom agreed.

**Fig. 2:**
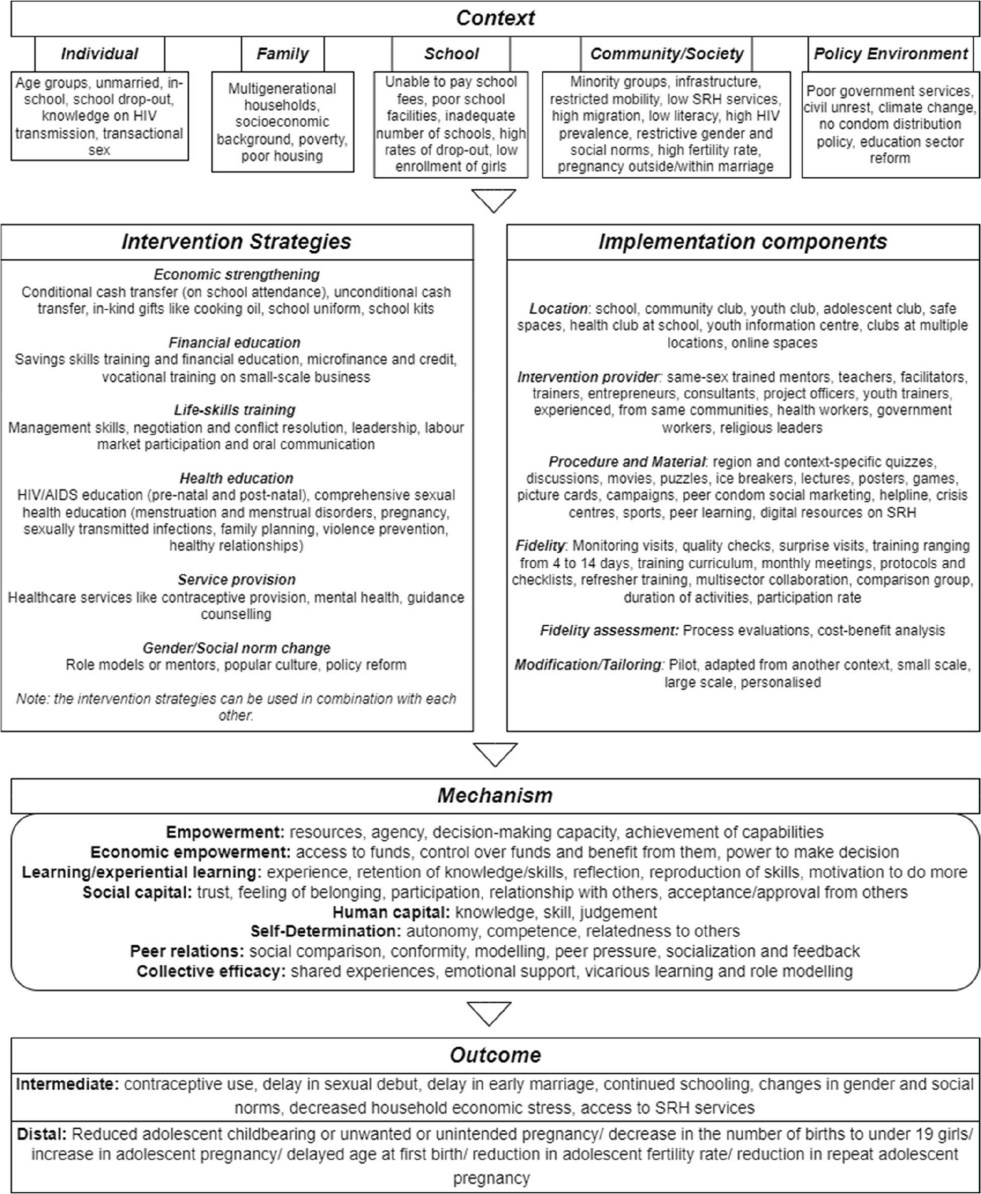
Initial intervention framework

**Fig. 3:**
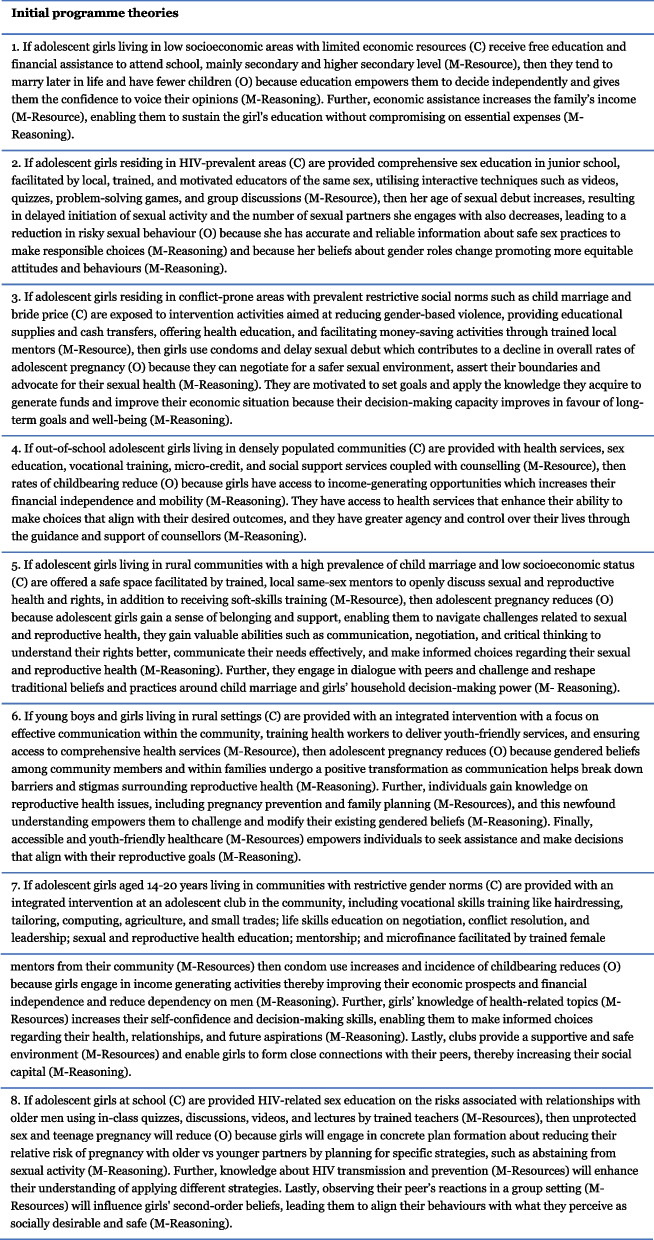
Initial programme theories

Using the codebook, we extracted data on the study context, intervention details, strategies, implementation techniques, outcomes, and possible mechanisms that explained how different intervention modalities from the studies retrieved in stage 1 may be linked to reducing adolescent pregnancy. We then mapped the extracted information onto an initial intervention framework, outlying the aggregated information with the outcome of interest (see Fig. 2). The context was divided into five categories based on the ecological framework [38], and the intervention was separated into two parts, intervention strategies, and implementation components (hereon called mechanism resources). The implementation components were organised using the TIDieR checklist [39]. The *mechanisms* and *outcomes* represent a composite of data presented in the intervention documents. The framework is only a representation of the granular overview of the components and will aid the development of IPTs [37, 40]. Based on the extracted CMO data and the framework, eight IPTs were proposed (Fig. 3). These IPTs were further revised by the review team and experts in two online meetings (questions for the experts can be found in the Additional file).

We observed that different resources were often used in combination with each other and elicited different mechanisms to impact adolescent pregnancy. For example, financial and life skill training in disadvantaged communities often used hands-on techniques in a safe space with peer interactions to elicit different mechanisms. While learning new life skills like negotiation or management increased the participant’s capability and motivated them to apply these newly acquired skills in daily life, learning financial skills triggered the accumulation of human capital and decreased dependence on men. These mechanisms may increase participants’ aspiration to look for alternatives to marriage and transactional sex and thereby decrease the risk of adolescent pregnancy. Another example is the economic strengthening applied in schools and communities in resource poor settings with high levels of poverty. These interventions integrated resources like interactive games and provided cash transfers to the family. For participants from socioeconomically low-income families with low literacy, cash transfers increased available funds, increasing their family’s flexibility to invest in education. Prolonged education triggered the increase in decision-making capacity and agency. Both these mechanisms can lead to a feeling of self-reliance and an increase in bargaining power and thereby lead to changes in risky sexual behaviour, delay marriage, and subsequently lower the risk of pregnancy. Figure 3 illustrates several pathways in which interventions may influence our outcome of interest. However, these are only the preliminary findings and will undergo further changes as the review progresses.

### Stages 2 and 3 — Search for evidence and appraisal of evidence

In the second stage, we developed a search strategy based on the critical components of the research question: adolescent population, adolescent pregnancy, gender transformative programmes, and LMICs (Additional Table 2). In this review, the primary outcome of interest — adolescent pregnancy — was defined per the threshold set by the World Health Organization as ‘pregnancy in a female adolescent under the age of 20’ [41]. This includes intended, unintended, and unwanted pregnancies. Further, we define gender transformative approaches as ‘programmes and interventions that foster critical examination of gender norms, create opportunities for individuals to actively challenge gender norms, promote gender equality, and address power inequities between persons of different genders’ [42]. All studies included in this review will be assessed based on this definition to be qualified as ‘gender transformative’ (see Fig. 1 of Additional file).

We will examine GTAs targeting the adolescent population (10–19 years) at any individual, group, or community level. In cases where the target population includes older participants, documents will be selected if results are disaggregated by age or if the majority of the sample comprises adolescents. All types of studies and documents describing interventions (i.e. non-experimental, experimental, quantitative, qualitative, mixed methods, implementation reports, project reports, policy briefs, blog posts) except systematic reviews, which examine a gender transformative intervention with adolescent pregnancy as one of the outcomes and that was conducted in an LMIC context, will be included. Interventions targeting both adolescent girls and boys as study participants will be considered. Only documents that provide a link between an intervention and the outcome of interest will be included. Documents published in English only will be included. The authors note that while translation software may support screening of non-English languages, data extraction in a non-English language is best conducted by a fluent speaker of that language, and the benefit of including them may be outweighed by the significant resources required [43].

The main aim of the systematic search is to identify additional evidence that will be eligible to refine, refute, or confirm the initial programme theories. The databases in our scope include (but are not limited to) the following: Embase, MEDLINE, ERIC, PsycINFO, CINAHL, Gender Studies Database, Reproductive Health Library, Studies in Family Planning, Reproductive Health Matters, International Family Planning Perspectives, and Population and Development Review. Additionally, grey literature will be included by locating resources on websites including OpenGrey, Advocates for Youth, Family Health International, Guttmacher Institute, Interagency Youth Working Group, International Center for Research on Women, Pathfinder International, Population Council, United Nations Population Fund, United Nations Children’s Fund, World Health Organization, and USAID. Based on realist methodology, the literature search process will be iterative and will embrace forward and backward citation tracking and contacting authors in cases where we need more information.

Using the above search strategy, we will pilot a standardised title and abstract screening form and full-text screening form to conduct a pilot exercise on a sample of 10 abstracts and 5 full-text articles to calibrate and test the review forms. Conflict will be mediated by another review author not involved in the screening process. We will use Rayyan, a free web tool designed to help researchers working on knowledge synthesis projects for deleting duplicates and conducting title and abstract screening [44]. Data on CMO configurations will be extracted using the codebook mentioned in stage 1, piloted by three authors using five articles to ensure codebook consistency. We will compare the results of the three authors and modify the codebook accordingly.

**Fig. 4:**
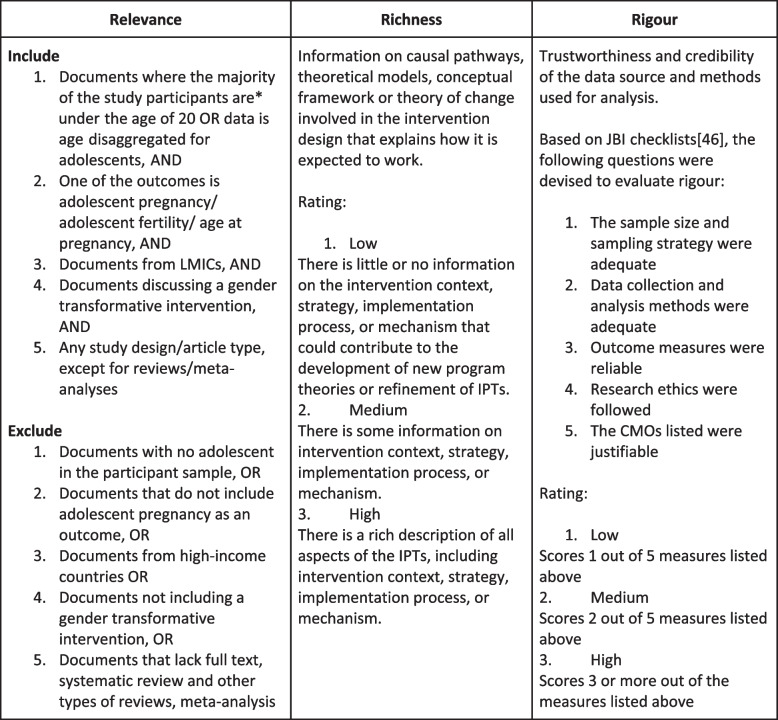
Appraisal tool — relevance, richness, rigour [46]

For the appraisal process, each article will be assessed based on relevance (whether it fits the inclusion criteria), richness (whether it can contribute to IPT building), and rigour (whether the information produced is credible and uses trustworthy methods) [32]. No article will be excluded or included based on just one criterion but on the overall value added to the research question (Fig. 4). The documents rated as ‘high’ according to our appraisal will be extracted first followed by those rated medium and low [45]. The authors will meet regularly to discuss their findings and modify the IPT.

### Stages 4 and 5 — Synthesise evidence to develop refined programme theory and disseminate recommendations

In the fourth stage, the extracted data on context, mechanism and outcome underlying the interventions of each document will be analysed. We will delineate CMOCs as ‘if…then…because’ statements. Similar CMOCs will be combined to construct richer CMOC statements, differentiating various contexts and mechanisms [47]. Finally, we will iteratively refine the earlier proposed IPTs into programme theories (PTs) by testing them against the comprehensive CMOCs. Each step of this process will be discussed by members of the review team. The PTs will be sent to the experts for their feedback, following which another cycle of refinement may be required. In the final stage, the refined programme theories will be shared with the broader audience via academic papers in open-access journals, conference and institutional presentations, and policy recommendations.

## Discussion

Gender transformative approaches seek to change the power dynamics that perpetuate gender inequality. They promote equitable gender roles and relationships and challenge harmful social norms reinforcing gender-based discrimination. They engage men and women to be agents of change instead of putting the onus on women alone. This realist review aims to demystify how these approaches work, for whom, and under which circumstances and implementation strategies to elicit positive health impact for adolescent girls. In the following steps, we will conduct a systematic search to collect evidence on our initial programme theories and further refine them. Refined programme theories based on the context-mechanism-outcome configuration will be the final product of this review.

Published literature on interventions to address adolescent pregnancy in LMICs does not adequately engage with the mechanisms behind the successful intervention. We still need to examine how gender transformative interventions measure changes in gender norms and if they have different results when targeting younger adolescents (10–14 years) vs older adolescents (15–19 years). As a result, the evidence is inconclusive and does not provide substantial guidance about how interventions for reducing adolescent pregnancy can be improved. Other gaps in the literature include a dearth of qualitative evidence and a lack of focus on interventions with boys [48]. In response to this, we will actively seek qualitative evidence, including implementation reports, process evaluations, monitoring reports, policy briefs, and other grey literature, along with studies that include boys in the intervention. We will also report if an intervention measured norm change and how that impacted the incidence of adolescent pregnancy. By doing so, we wish to understand what adolescents think and how they react to intervention procedures, thus informing programme design further. Overall, this research will be helpful for policymakers and programme designers to understand the importance of considering the context, intervention resources, and related mechanisms when designing programmes to address adolescent pregnancy.

### Supplementary Information

**Additional file 1:** **Supplementary tables. Table 1.** Codebook. **Table 2.** Search Strategy. **Supplementary Figure 1.** Definition of gender continuum for interventions

## Data Availability

Not applicable.
