# Supplementary material for: Mechanisms behind gender transformative approaches targeting adolescent pregnancy in low- and middle-income countries: a realist synthesis protocol
Source: Syst Rev. 2024 Mar 23;13:95. doi: 10.1186/s13643-024-02513-4 (PMC10960499; doi:10.1186/s13643-024-02513-4)
Supplement: Supplementary file 1 — Additional file 1: Supplementary tables. Table 1. Codebook. Table 2. Search Strategy. Supplementary Figure 1. Definition of gender continuum for interventions [file 13643_2024_2513_MOESM1_ESM.docx]

# Mechanisms behind gender transformative approaches targeting adolescent pregnancy in low- and middle-income countries: a realist synthesis protocol

**Shruti Shukla***^§^*, MSc, TUM School of Social Sciences and Technology, Technical University of Munich, Germany

**Ibukun-Oluwa Omolade Abejirinde**, MD, PhD, Division of Social & Behavioural Health Sciences, University of Toronto Dalla Lana School of Public Health & Women’s College Hospital Research Institute, Toronto, Canada.

**Sarah R. Meyer**, PhD, Institute for Medical Information Processing, Biometry, and Epidemiology, Ludwig-Maximilians-Universität München

**Yulia Shenderovich**, PhD, Wolfson Centre for Young People’s Mental Health; Centre for Development, Evaluation, Complexity and Implementation in Public Health Improvement (DECIPHer), School of Social Sciences; Cardiff University, United Kingdom

**Janina Isabel Steinert**, DPhil, TUM School of Social Sciences and Technology, Technical University of Munich, Germany

*^§^corresponding author*

Richard-Wagner-Straße 1

80333 München, Germany

+49 151 290 247 10

Shruti.shukla@tum.de

# Additional file

## Table 1: Codebook

| **Study details** | | | | | | | | | | |
| --- | --- | --- | --- | --- | --- | --- | --- | --- | --- | --- |
| **Study ID** | **Publication type** | **Publication year** | **Geographic area** | **Target population** | **Target Age** | **Sampling** | **Sample characteristics** | **Sample size (only adolescents)** | **Type of study** | **Aim of study** |

| **Intervention** | | | | | | |
| --- | --- | --- | --- | --- | --- | --- |
| **Intervention name** | **Aim** | **Intervention Strategy (e.g. education, skills training, communication campaign etc.)** | **Details/ Activities/ Content** | **Duration/ Frequency** | **Adaptation (from another intervention)** | **Description of staff, their training, their supervision** |

| **Context** | | **Mechanism** |
| --- | --- | --- |
| **Setting (i.e. community, school etc; region characteristics)** | **Individual characteristics (participant's family/HH background, economic back. etc)** | **Theoretical or hypothesised in the intro/ methods/ discussion/ results)** |

| **Outcome** | | | |
| --- | --- | --- | --- |
| **Type of outcomes** | **Measures (Outcome of interest)** | **Time frame of measures** | **Findings (including adverse effects)** |

## Table 2: Search Strategy

| **Number** | **Criteria** | **Terms** |
| --- | --- | --- |
| 1 | Adolescent | adolescent* OR teen* OR young people OR youth* OR school age* OR juvenile* OR minor OR minors OR youngster* OR underage* OR teenager* OR emerging adult* OR early adulthood OR young adult* OR young women OR young men OR boy OR boys OR girl OR girls |
| 2 | Adolescent  pregnancy | Adolescent pregnancy OR teen pregnancy OR teenage pregnancy OR young maternal age OR early pregnancy OR unintended pregnancy OR unwanted pregnancy OR adolescent childbearing OR adolescent motherhood OR teenage motherhood OR teenage childbearing OR young maternal health OR adolescent fertility |
| 3 | Gender transformative interventions | GTP OR GTI OR gender transformative interventions OR gender transformative programs OR gender transformative approaches OR GTA OR gender transformative initiative OR gender program OR gender intervention OR gender project OR gender inequality OR gender norms OR gender OR gender club OR gender training OR power inequity |
| 4 | Low- and middle-income countries (LMICs) | Afghanistan OR Albania OR Algeria OR Angola OR Antigua OR Barbuda OR Argentina OR Armenia OR Armenian OR Aruba OR Azerbaijan OR Bahrain OR Bangladesh OR Barbados OR Benin OR Byelarus OR Byelorussian OR Belarus OR Belorussian OR Belorussia OR Belize OR Bhutan OR Bolivia OR Bosnia OR Herzegovina OR Hercegovina OR Botswana OR Brasil OR Brazil of Bulgaria OR Burkina Faso OR Burkina Fasso OR Upper Volta OR Burundi OR Cambodia OR Khmer Republic OR Kampuchea OR Cameroon* OR Cameron OR Camerons OR Cape Verde OR Central African Republic OR Chad OR Chile OR China OR Colombia OR Comoros OR Comoro Islands OR ComORes OR Mayotte OR Congo OR Zaire OR Costa Rica OR Cote dIvoire OR Ivory Coast OR Croatia OR Cuba OR Cyprus OR Czechoslovakia OR Czech Republic OR Slovakia OR Slovak Republic OR Djibouti OR French Somaliland OR Dominica OR Dominican Republic OR East Timor OR East Timur OR Timor Leste OR Ecuador OR Egypt OR United Arab Republic OR El Salvador OR Eritrea OR Estonia OR Ethiopia OR Fiji OR Gabon OR Gabonese Republic OR Gambia OR Gaza OR Georgia Republic OR Georgian Republic OR Ghana OR Gold Coast OR Greece OR Grenada OR Guatemala OR Guinea OR Guam OR Guiana OR Guyana OR Haiti OR Honduras OR Hungary OR India OR Maldives OR Indonesia OR Iran OR Iraq OR Isle of Man OR Jamaica OR Jordan OR Kazhakstan OR Kazakh OR Kenya OR Kiribati OR Korea OR Kosovo OR Kyrgystan OR Kirghizia OR Kyrgyz Republic OR Kirghiz OR Kirgizstan OR Lao PDR OR Laos OR Latvia OR Lebanon OR Lesotho OR Batusoland OR Liberia OR Libya OR Lithuania OR Macedonia OR Madagascar OR Malagasy Republic OR Malaysia OR Malaya OR Malay OR Sabah OR Sarawak OR Malawi OR Nyasaland OR Mali OR Malta OR Marshall Islands OR Mauritania OR Mauritius OR Agalega Islands OR Mexico OR Micronesia OR Middle East OR Moldova OR Moldovia OR Moldovian OR Mongolia OR Montenegro OR Morocco OR Ifni OR Mozambique OR Muanmar OR Myanma OR Burma OR Namibia OR Nepal OR Netherlands Antilles OR New Caledonia OR Nicaragua OR Niger OR Nigeria OR Northern Mariana Islands OR Oman OR Muscat OR Pakistan OR Palau OR Palestine of Panama OR Paraguay OR Peru OR Philippines OR Philipines OR Phillipines OR Phillippines OR Poland OR Portugal OR Puerto Rico OR Romania OR Rumania OR Roumania OR Russia OR Russian OR Rwanda OR Ruanda OR Saint Kitts OR St Kitts OR Nevis OR Saint Lucia OR St Lucia OR Saint Vincent OR St Vincent OR Grenadines OR Samoa OR Samoan Islands OR Navigator Island OR Navigator Islands OR Sao Tome OR Saudi Arabia OR Senegal OR Serbia OR Montenegro OR Seychelles OR Sierra Leone OR Slovenia OR Sri Lanka OR Ceylon OR Solomon Islands OR Somalia OR South Africa OR Sudan OR Suriname OR Surinam OR Swaziland OR Samoa OR Syria OR Tajikistan OR Tadzhikistan OR Tadjikistan OR Tadzhik OR Tanzania OR Thailand OR Togo OR Togolese Republic OR Tonga OR Trinidad OR Tobago OR Tunisia OR Turkey OR Turkmenistan OR Turkmen OR Uganda OR Ukraine OR Uruguay OR USSR OR Soviet Union OR Union of Soviet Socialist Republics OR Uzbekistan OR Uzbek OR Vanuatu OR New Hebrides OR Venezuela OR Vietnam OR Viet Nam OR West Bank OR Yemen OR Yugoslavia OR Zambia OR Zimbabwe OR Rhodesia OR developing countr* OR less* developed countr* OR under developed countr* OR underdeveloped countr* OR middle income countr* OR low* income countr* lmic OR lmics OR low income countr* OR middle income countr* OR low and middle income countr* |

Databases/Journals:

EBSCO, ProQuest, Web of Science, Scopus, EconLit, Gender Studies Database, Global Health, Medline, Development Experience Clearinghouse, Reproductive Health Library, International Family Planning Conference, Studies in Family Planning, Reproductive Health Matters, International Family Planning Perspectives, and Population and Development Review.

Grey lit

Advocates for Youth, Family Health International, Guttmacher Institute, Interagency Youth Working Group, International Center for Research on Women, International Planned Parenthood Federation, Joint United Nations Program on HIV and AIDS, Marie Stopes International, Pathfinder International, Population Council, United Nations Population Fund, United Nations Children’s Fund, World Health Organization, USAID.

## Figure 1: Definition of gender continuum for interventions


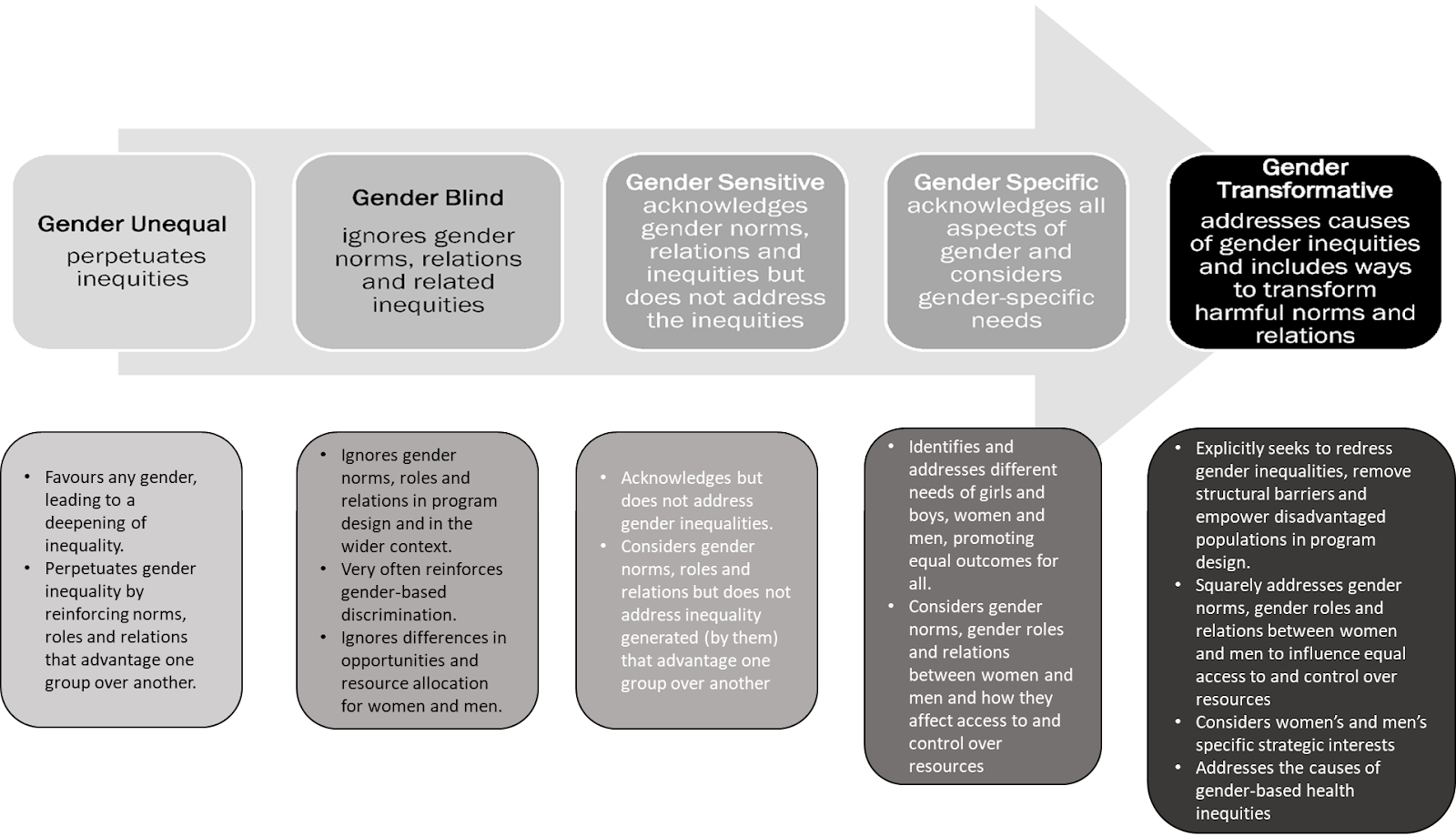


Expert Consultation questions (Round 1 – IPT)

Based on your experience in the implementation of science/ sexual and reproductive health programs:

1. Are the context categories mentioned in the initial program theory relevant to gender transformative interventions? What else would you add to these categories that we still need to consider? Or What would you delete, given they are not as important? Or would you suggest a different set of categories?
2. What do you think about the intervention strategies and techniques mentioned in the flowchart? Are they well suited to impact adolescent pregnancy? When will they make the maximum impact? Will you add another strategy/technique or delete any of them, why?
3. What is your opinion on how a gender transformative intervention might cause its outcomes? How do you think the mentioned IPTs might cause or help to cause a reduction in adolescent pregnancy?
4. What do you think about the proposed mechanisms in the IPTs? Which mechanisms, according to you, are the most important in bringing long-lasting behaviour change?
5. Do you think the outcomes will be the same for all adolescents (boys, girls)? In what ways will they be different?
6. If you could change something about the IPTs to make it more relevant or understandable, what would you change and why?
